# Caring for children with disabilities in Kilifi, Kenya: what is the carer's experience?

**DOI:** 10.1111/j.1365-2214.2010.01124.x

**Published:** 2011-03

**Authors:** J K Gona, V Mung'ala-Odera, C R Newton, S Hartley

**Affiliations:** * Centre for Geographic Medicine Research (Coast), Kenya Medical Research InstituteKilifi, Kenya; † Neuroscience Unit, Institute of Child Health, University College LondonLondon; ‡ Clinical Research Unit, London School of Hygiene and Tropical MedicineLondon; § University of East AngliaNorwich, Norfolk, UK

**Keywords:** carers, challenges, coping strategies, disability

## Abstract

**Background:**

Carers of children with disabilities have repeatedly highlighted their feelings of discrimination, stigma and exclusion in many domains of their lives. There is little research from Africa addressing these issues. This study investigated the challenges encountered by these carers and the mechanisms of coping with these challenges while caring for children with disabilities in a poor rural setting in Kenya.

**Methods:**

Thirty-five in-depth interviews were conducted with 20 carers, 10 community members and 5 primary school teachers. Ten unstructured observations were also conducted in home environments to observe mechanisms used in meeting the needs of the children with disabilities. All interviews were tape-recorded, transcribed and translated from the local dialect. Note-taking was performed during all the observations. Data were stored in NVivo software for easy retrieval and management.

**Results:**

The arrival of a disabled child severely impairs the expectations of carers. Hospital staff underestimate carers' emotional distress and need for information. Fear for the future, stress, rumour-mongering and poverty are encountered by carers. As they grapple with lost expectations, carers develop positive adaptations in the form of learning new skills, looking for external support and in some cases searching for cure for the problem. For their emotional stability, carers apply spiritual interventions and sharing of experiences.

**Conclusion:**

Despite the challenges faced by the carers, values and priorities in adaptation to the challenges caused by the child's disability were applied. It is recommended that these experiences are considered as they may influence programmes that address the needs of children with disabilities.

## Introduction

### Challenges

In the West, carers of children with disabilities have repeatedly highlighted their feelings of discrimination, stigma and exclusion (Corrigan *et al*. 2003; Rosenzweig & Huffstutter 2004; Huffstutter *et al*. 2007). There is little research from Africa addressing these issues. Studies from Uganda reveal that carers of children with disabilities, who are mainly mothers or grandmothers, are subjected to stress in the form of physical ailments, isolation and insufficient time for other household chores (Bwana & Kyohere 2001; Hartley *et al*. 2004). Carers of children with hearing or speech deficits face challenges of a breakdown in communication because of inadequate knowledge in the use of signs (Hartley *et al*. 2004).

Existing research has shown that carers of children with special needs undergo strain because of unusual demands that include disrupted family and social relationships, exhaustion, financial difficulties, guilt and parenting distress (Angold *et al*. 1998; Yatchmenoff *et al*. 1998; McDonald *et al*. 1999). Although not much has been reported on the economic consequences of caring for a child with disability, there are studies that have examined direct costs of care, and the indirect effects on families' financial position. Families with children who have mental health conditions have reported substantial costs incurred by treatment (Burns *et al*. 1995; Ringel & Sturm 2001). Outpatient care accounts for most of the expenditure by the families. The indirect costs include limited career aspirations and time diverted from the ordinary activities of daily living to the needs of the disabled child (Scott *et al*. 2001; Lynch 2007).

The association between care-giving and negative health outcomes has been investigated in many western studies. In a study conducted in Canada, Cadman (1991) established that parents of children with disabilities were more likely to experience depression and distress than parents of children without disabilities. Dyson (1993) in a study in USA reported significant increased parental stress in parents of children with disabilities. These were also found to be pessimistic with regard to their future (Dyson 1993). In another American study of mothers of children with disabilities, poor emotional and physical health was reported, further, the mothers experienced greater demands on their time by the child (Dunst *et al*. 1986). Similarly, other studies have reported that mothers of children with disabilities had more difficulty caring for their children and felt lack of time for themselves because of increased daily demands associated with the caring for a child with a disability (Friedrich & Friedrich 1981; Gowen *et al*. 1989).

### Coping strategies

Evidence suggests that there is considerable variation in how carers adapt to their care-giving demands (Solomon *et al*. 2001; White & Hastings 2004; Rosenzweig & Huffstutter 2004). Contextual factors such as socio-economic status, severity of disability and behavioural problems of child, social support and coping strategies have been associated with psychological and/or physical outcome (Trivette *et al*. 1996; Brannan *et al*. 1997; Angold *et al*. 1998; McDonald *et al*. 1999). A number of studies from western countries have shown that spousal support or satisfaction with marital relationship is associated with lower levels of stress in parents of children with disabilities (Kazak & Marvin 1984; Sloper *et al*. 1991; Herman & Thompson 1995). Support from extended family members, especially grandparents has the potential of helping parents cope a disability (Hastings 1997).

In Africa, the phenomenon of extended family is disintegrating because of many causes including poverty and HIV/AIDS pandemic (Hartley *et al*. 2004). However informal sources of support such as friends and religious groups and the use of respite care service accorded to carers have been associated with reduced stress in carers of children with disabilities (Chan & Sigafoos 2001; Hastings & Johnson 2001, Smith *et al*. 2001; Salovita *et al*. 2003; Hartley *et al*. 2005). But carers have reported reduced availability of these informal sources of support when the degree of disability in the child is severe (Shin & McDonaugh 2008).

High levels of family cohesion and togetherness has been identified as an important coping mechanism (White & Hastings 2004). In Africa, ethnic and cultural bonding has been highlighted in different societies. The strength of the African societies lies in their cohesiveness and supportive nature in all aspects of life. However this cohesiveness has been compromised because of the collapse of traditional systems coupled with social inequality existing in most African communities (Nduati 1995).

Building relationships with others in a similar situation has been identified as a key indicator of coping ability among carers (Solomon *et al*. 2001). Kagan *et al*. (2008) found out that participation in community-based peer support networks may bring enrichment and a sense of psychological belonging to the lives of parents of a child with a disability. Several societal trends that include the shift from institutional care to community and home-based care, and the growing interest in family centred services have led to the growing interest in support groups. This paradigm shift has not been practical in African communities. Studies in Uganda and Kenya have revealed that disability is still perceived as a curse, punishment from God for wrong doing, or work of evil spirits (Hartley *et al*. 2004; El Sharkawy *et al*. 2006; Gona *et al*. 2006). This has resulted in parents hiding their children with disabilities for fear of isolation, segregation or discrimination.

The perception of disability in some African communities has stereotype and negative implications to the carers. The physical and psychological consequences during their care-giving processes, and their adaptive coping strategies applied have not been explored in-depth in African communities. This impairs the development of disability intervention programmes for children with disabilities because interventions based on carers' experiences would have the potential of facilitating support and engagement of the community. This study investigated these experiences in a rural African community with the purpose of identifying important issues to inform good disability intervention practice.

## Design and methodology

### Study design

We employed a qualitative phenomenological approach to explore the lived experiences of carers of children with disabilities. The focus of the study was on the challenges and coping strategies. In-depth interviews and unstructured observations were conducted.

The study addressed two research questions:

1. What challenges do carers of children with moderate and severe disabilities in Kilifi face?
2. How do they cope with these challenges?

### Sample size determination and sampling procedure

Children for the study were selected from 104 children identified in a house-to-house neurological survey of children aged6–9 years using the ten-question questionnaire (TQQ) conducted in Kilifi, Kenya (Mung'ala-Odera *et al*. 2004). Carers of the selected children were the study participants. Twenty children with moderate and severe disability in cognition, motor, epilepsy, hearing and vision were purposively selected from the 104 children identified in the survey. Definitions of moderate and severe disability were adopted from the World Health Organization procedure manual (Table 1). The 104 children were first divided according to their impairment groups as follows: 18 children with cognitive impairment, 25 with hearing impairment, 8 with visual impairment, 18 with motor impairment and 35 with active epilepsy. A purposive sample of four children from each group of impairment was selected. Inclusion criteria included gender, age, geographical locations and proximity from health facilities (Table 2).

**Table 1:** Definition of moderate and severe impairment [adopted from WHO procedure manual (Mung'ala-Odera *et al*. 2004)]

Impairment	Moderate	Severe
Cognitive	*Z*-score of below −2 on two or more of the seven tests OR mean *Z*-score for all tasks below −2 for a child who had performed construction task and a non-verbal task but not the key verbal tasks (picture vocabulary and information questions tasks OR the mean *Z*-score for verbal tasks was below −2)	*Z*-score of below −3 on two or more of the seven tests OR mean *Z*-score for all tasks below −3 for a child who had performed construction task and a non-verbal task but not the key verbal tasks (picture vocabulary and information questions tasks OR the mean *Z*-score for verbal tasks was below −3)
Motor	Difficulty in holding implements, dressing and sitting upright. Able to move around with help	Inability to walk and absence of functional use of hands
Epilepsy	More than one non-febrile seizure per month	More than one non-febrile seizure per week
Hearing	A 41–70 dB loss in the best ear and difficulty in hearing even with a hearing aid	More than 70 dB loss in the best ear, no useful hearing
Vision	Vision loss of 6/18	Visual acuity worse than 6/60 meters, only light perceptions

**Table 2:** Distribution of children

	Cognitive impairment	Hearing impairment	Vision impairment	Motor impairment	Epilepsy	Total
Male	2	2	2	2	2	10
Female	2	2	2	2	2	10
Urban	2	2	2	1	2	09
Rural	2	2	2	3	2	11
Near	2	2	3	2	2	11
Far	2	2	1	2	2	09
Total sample	4	4	4	4	4	20

Near health facility: 0–5 Km; far from health facility: above 5 Km.

Out of the 20 carers, 15 consented to participate in the study. We then purposively recruited another five participants who consented. Each carer purposively identified a community member they trusted to take part in the study. Ten community members out of the 20 named by the carers consented to be interviewed. All five teachers of the selected five children attending school were comprehensively sampled.

### Development of research tools

A checklist of questions was developed by the research team through discussion and consensus. These questions were piloted with mothers of children with disabilities attending occupation therapy at the district hospital (Table 3).

**Table 3:** Interview schedule

**For carers**
1. What do you go through in your day-to-day caring of X?
2. How do these tasks affect you?
3. Why do you think they are happening to you?
4. How do you cope?
5. How do you get assistance?
**For community members and teachers**
1. What does (carer's name) go through in her/his day-to-day caring of X?
2. How do these affect him/her?
3. Why do you think such things are happening to him/her?
4. How does he/she cope?
5. How does he/she get assistance?

### Methods of data collection

#### In-depth interviews

In-depth interviews were conducted using open-ended questions to allow individual variation as described by Patton (1990). Three groups of study participants: carers, community members, and teachers were interviewed by JG. JG is a native of Kilifi District and conversant with the languages and culture of the people. He is experienced in the interview technique. Both the carers and the community members were interviewed at their homes and the teachers at school. Guiding questions focused on daily challenges and coping strategies applied by the carers (Table 3). The interviews were tape-recorded, transcribed and then translated by JG. The interviews were randomly monitored by a senior fieldworker working with Kenya Medical Research Institute (KEMRI) through observations to assure consistency in interview techniques for maintaining the validity of the information collected.

### Observations

Passive observations consisted of systematic watching of behaviour and talk directed to the child from the carer or sibling to capture the unexpected, unusual or unsaid and provide knowledge of the context in which events occur (Patton 1990). Both verbal and non-verbal cues were monitored during the observations by a skilled observer. These direct passive observations were carried out with 15 children in their homesteads for duration of 1 h per observation. The five children in school were not observed as observations were scheduled to be conducted in home environments. During the observation, note-taking on verbal and non-verbal cues was done.

### Data analysis

Inductive analysis as described by Patton (1990) was utilized. The interviews were transcribed and translated in word document, then imported into NVivo programme for storage, easy retrieval and management. This process was applied to the raw data from the observation notes. With the assistance of NVivo, patterns and themes of analysis were formulated. During the coding process free nodes were first created. Then patterns were identified and put together to form tree nodes each bearing a name of a theme. Once the thematic structure was established all the data was coded to this structure.

Data triangulation from the interviews and the observations was done for validation and reliability. The recording equipment was checked regularly to ensure it was functioning well. The interview transcripts and observation notes were shared and cross-checked by co-researchers.

### Ethical consideration

The study was approved by the Communication and Consent Committee in Kilifi and the National Ethics Review Committee in Nairobi (SSC #1235). The study participants signed an informed consent before taking part in the study.

## Results

The results are presented in two main sections: (1) challenges faced by carers and (2) coping strategies.

This study used the definition of coping by Lazarus & Folkman (1984) as ‘the constantly changing cognitive and behavioural efforts to manage specific external and/or internal demands that are appraised as taxing or exceeding the resources of a person’. They categorize coping to have two dimensions: problem-focused, which is directed at managing or altering the problem that brings the distress, and emotional-focused that is directed to regulating the emotional response to the problem.

### 1Challenges faced by carers

Two main themes emerged from the data: shattered dreams and expectations from healthcare staff. Shattered dreams main theme was broken down into four subthemes: fear for future, stress; rumour-mongering and poverty. Expectations from health staff had one subtheme, lack of information.

#### Shattered dreams

The data show that carers undergo pain and devastation when they realize that their future dreams and expectations would not be met because of the child with disability. Most families bank on their children for future prosperity and well-being. Parents have to clothe them, feed them and meet all their daily needs with less prospects of maximum output from the child. The expectation that the child would marry is reduced.

You get a child to help you in future. So if those children you term will help you in future have no future. … wash them, take them to bed. They will never help me. [Mother (MI), rural, far from health facility]

One of the main outcomes of a child's disability is carer's stress. The data reveal stress in the form of insufficient time for other chores and responsibilities and isolation from community activities because of time spent attending to the child at home.

I cannot help myself to earn a living since I have to look after them because they cannot walk or sit and my ways to succeed in life are always closed. [Mother (MI), rural, close to health facility]

… only that I can't go out to perform other duties or attend to other issues rather than staying at home caring for her. [Grandmother (CI), far from health facility]

The data further reveal that it is a common aspect for community members to speculate about the cause of a child's disability. The data indicate that because of the complexity of possible causes of severe disability, coupled with cultural beliefs and superstitions related to disability, people engage in rumour-mongering. The carer is either associated with evils spirits (jinnis), punishment from God, or witchcraft.

So bringing up such a child people look at you badly … as if there is something wrong you did … something bad that you committed that is why you had a child like that. [Mother (CI), urban, close to health facility]

When a person gets a disabled child, people think of witchcraft. [Community member (HI) rural, far from health facility]

Because of poverty carers become handicapped in providing optimal care to the children. All the external resources available to facilitate good caring practices are reduced by poverty. The data reveal a lot of difficulties among carers in meeting basic needs like food, clothing, fees and money for drugs.

So there are other important things they would do to this child but cannot be done because of the poor condition they are in. [Teacher (HI), rural, close to health facility]

The family is poor. This forces the family to undergo more expenses to make child get the right education, clothing and food. [Community member (MI), rural far from health facility]

#### Expectations from healthcare staff

The data suggest that most carers expected the medical staff to give information about the child's condition that they could easily understand. However, information given was scanty or sometimes none at all.

I took the child to Kilifi Hospital, but I was told the hospital does not attend to children like that. That was not enough information to me. [Mother (CI), rural, far from health facility].

Then when she came from hospital disappointed. She was not attended at the hospital. I don't know about her I think she went to a traditional healer. [Teacher (HI), rural, close to health facility]

The observational data support all the themes. Carers seemed be lonely and without hope, as they were observed seated alone with one hand on the cheek. According to the Giriama culture, one observed seated in that position signifies helplessness. During feeding, carers of children with severe disabilities were observed to get frustrated. This was expressed in the form of leaving the food in front of the child and walking away. Poverty was observed in the standard of the housing, number of meals eaten per day, the quantity of food served and lack of livestock at the homestead. Most houses were made of mud with half thatched roofs. Livestock were rarely noticed in most of the homesteads visited.

### 2Coping strategies

This section had two main themes: problem-focused and emotional-focused. Learning new skills, search for cure and external support were the subthemes for problem-focused. Emotional-focused had two subthemes: divine interventions and sharing of experiences.

#### Problem-focused

The data indicate that because of a lot of care-giving strain and lack of rehabilitation services in the community, carers had to learn new skills to cope with child's disability. They improvised materials for exercises at their homes in order to maintain continuity of therapy.

… I had learnt the exercises which are being provided at the hospital. So I continued giving her the exercises at home till she started walking. [Mother (MI), rural, far from health facility]

I used to make a hole to support my child sit [Mother (CI), rural, close to health facility]

External support was in the form of materials. Non-governmental organizations, charitable organizations and sometimes individuals supported the carers in providing wheelchairs and food. Sometimes assist in paying treatment bills.

Another help was from the Port Reitz people. They brought those wheelchairs and food. [Mother (PI), rural, close to health facility]

I benefit from organisations that have assisted us like where I sent him to get treatment … had promised me they would perform an operation. [Grandmother (VI), rural, close to health facility]

The data suggest that most carers engage in looking for alternatives with the hope of getting a cure. The obsession that there could be a cure somewhere facilitates the need to move from healer to healer including traditional healers (‘Mganga’).

… was another traditional healer (mganga) who was good, she should go and try that because maybe the child was bewitched. She could be lucky and get healed. [Community member (E), rural, far from facility]

That is where she was really treated traditionally. All the suggested methods were performed, every thing. [Mother (CI), rural, close to health facility]

The data reveal that sometimes it became necessary for carers to seek for confirmation or second opinions of what steps to take from divine tellers. Divine tellers revealed the causes of the disability, type of treatment to be offered and preventive measures for re-occurrence.

Then she again became sick went to a diviner and was told that she was bewitched. [Grandmother (VI), rural, far from health facility]

First a diviner (diviner tells causes of sickness and procedures of treatment) was consulted. Then all items (TASA) suggested by the diviner were collected including a hen. [Mother (MI), rural, close to health facility]

#### Emotional-focused

The data indicate that carers sort spiritual interventions for emotional satisfaction. Carers who turned to God and became ‘born-again’ believed that God had a purpose for that child to have a disability. There is evidence from the data of children with disabilities being taken to churches for deliverance prayers or for God to see and sympathize with their situation.

But if it was your wish that he will live, then that is upon you. But if is the wish of the devil, he will be defeated in the name of Jesus. I decided to be born-again because of that child. [Mother (MI), rural, close to health facility]

We were given this child by God, so take her back to God … to church for prayers. [Community member (CI), rural, close to health facility]

Sharing of experiences with one another was highlighted in the data as a mode of meeting emotional demands. Carers of children with disability talked to each other, shared experiences and advised each other on how best to cope with the child.

We call parents together to share their experiences. This gives them a lot of emotional support. [Teacher (VI), urban, close to health facility]

When I met her in the bus I knew that this one had the same problems as I had. We asked each other and it was true we were heading for the same issue. [Mother (MI), rural, far from health facility]

## Discussion

A main issue that emerged from the data is that carers of children with disabilities had lost dreams and aspirations. This is particularly important in African societies as people expect their children to provide and support them during old age. When a disabled child is born the future becomes uncertain. Carers engage in different activities to address this uncertainty, but when positive outcome is not realized, they wonder if it was worth the effort.

One of the conclusions to be drawn from the data is that carers in Kilifi who participated in this study expressed elements of stress as they struggled to meet the needs of the child. A mother needed time to attend to community obligations. But most of her time was spent on the child with a disability. Sometimes the ability of the carer to look for means of living for the family is significantly affected by the burdens of care. This leads to lack of basic needs for other siblings leading to more stress on the carer.

Speculation about the child's disability among community members could possible result from lack of proper information on what causes a disability. As previous studies in Kilifi have found that disability is associated with evil spirits, punishment from God or witchcraft, this could possible explain the aspect of people spreading rumours. Carers expressed feelings of guilt about their child's disability leading to low esteem and feelings of helplessness.

An impediment to the caring process expressed by most participants was poverty. Taking into account that 60% of people in Kilifi District live below the poverty line (Kenya Bureau of Statistics 2003), this could possible explain why carers of children with disabilities are hard hit by poverty. Because poverty leads to disability and disability likewise leads to poverty, this could be a big challenge to carers as they could never have enough resources for anything.

Another point of discussion that emerged from the analysis is that hospital staff underestimated carers' emotional distress and need for information. Participants interviewed indicated that carers visit health facilities with their disabled child, but the reception accorded to them in these facilities are not to their expectations. Carers experience frustration as they are detained in hospital wards without any medical attention or advice. Sometimes carers left the hospital without proper discharge procedures. This attitude from the hospital staff could probably explain why most carers preferred attending a traditional healer than a hospital clinician.

Finally the data indicate that carers adapt different mechanisms to cope with the antagonizing forces because of the disabled child. Participants said that when carers found themselves in a state of helplessness, they engaged in spiritual beliefs. Hope and spiritual beliefs are important factors in people's ability to meet life challenges. They have been found to be important protective factors to parents of children with disabilities (Poston & Turnbull 2004).

## Conclusion

Despite the challenges faced by the carers, values and priorities in adaptation to the challenges caused by the child's disability were applied. It is recommended that these experiences are considered as they may influence programmes that address the needs of children with disabilities.

**Key messages**

- Disability of a child shatters the dreams of a family.
- Inadequate information from health personnel given to carers.
- Carers engage in looking for alternative Treatment.
- Spiritual intervention is sort for emotional satisfaction.
